# On the Fidelity of NS-3 Simulations of Wireless Multipath TCP Connections

**DOI:** 10.3390/s20247289

**Published:** 2020-12-18

**Authors:** Monika Prakash, Atef Abdrabou

**Affiliations:** Department of Electrical Engineering, UAE University, Al-Ain, Abu Dhabi PO 15551, UAE; 201790521@uaeu.ac.ae

**Keywords:** multipath TCP, ns-3, simulation, direct code execution, congestion control, heterogeneous, wireless networks, Wi-Fi, LTE, 5G

## Abstract

The multipath transmission control protocol (MPTCP) is considered a promising wireless multihoming solution, and the 3rd generation partnership project (3GPP) includes it as a standard feature in the fifth-generation (5G) networks. Currently, ns-3 (Network Simulator-3) is widely used to evaluate the performance of wireless networks and protocols, including the emerging MPTCP protocol. This paper investigates the fidelity of the Linux kernel implementation of MPTCP in the ns-3 direct code execution module. The fidelity of MPTCP simulation is tested by comparing its performance with a real Linux stack implementation of MPTCP using a hardware testbed for two different setups. One setup emulates the existence of a bottleneck link between the sending and receiving networks, whereas the other setup does not have such a bottleneck. The fidelity of ns-3’s simulation is tested for four congestion control algorithms, namely Cubic, linked-increases algorithm (LIA), opportunistic LIA (OLIA) and wVegas for relatively short and long data flows. It is found that the uplink MPTCP throughput performance exhibited by the ns-3 simulator matches the hardware testbed results only if the flows are long-lived and share no common bottleneck link. Likewise, the MPTCP throughput achieved during a downlink scenario using the ns-3 simulator and the hardware testbed are close to each other across all algorithms except wVegas regardless of the flow size if there is no bottleneck link. Moreover, it is observed that the impact of LTE handover on MPTCP throughput is less significant in the simulator than the real hardware testbed, and it is setup-dependent.

## 1. Introduction

There has been tremendous growth in wireless data traffic and the number of wirelessly connected devices in recent years. The main goals of 5G networks, such as high data rates and low latency, can be supported by millimeter wave (mmWave) technology. However, mmWave communication signals lack strong diffraction and are more prone to blockages by the physical objects in the environment, which may result in the disruption of communication links [1,2]. Even the human body is considered a strong blocker of the mmWave communication system. In fact, there can be self-body blockages that are caused by the users of the communication device themselves [3]. The authors of [4] showed that the human body can bring down the mmWave signal strength by around 20 dB and block the link for approximately 500 ms. The frequent blockages of mmWave connections and long blockage duration can significantly impact 5G communication reliability. Hence, it is essential to support redundancy in network communication links. The availability of multiple network connections provides a failover solution to compensate for a blockage-affected mmWave link by using another communication link. Luckily, the concept of wireless multihoming is gaining higher traction, driven by emerging heterogeneous wireless networks and the increased availability of wireless devices equipped with multiple wireless interfaces. The simultaneous connection to two networks is standardized by 3GPP as Dual Connectivity in Release 12 [5]. The 5G networks are designed to integrate multiple radio access technologies, such as cellular and Wi-Fi, resulting in multiple radio access technologies (RAT) environment. Multihoming is therefore considered to be one of the key features of the 5G networks.

Multipath TCP is a transport layer protocol designed for networks with multihomed devices. Since the legacy transport layer protocol of the Internet, TCP (transmission control protocol), supports only single-path connections, the Internet engineering task force (IETF) introduced MPTCP (backward compatible with TCP) to support multipath connections [6]. The extension of the standard TCP to support multiple paths simultaneously is expected to increase the overall resource utilization and, in turn, the network throughput. Moreover, the availability of multiple paths to MPTCP provides resilience to network failure. Furthermore, 3GPP recently standardized MPTCP as an integral feature of 5G in Release 16 [5]. Thus, multipath TCP is gaining research focus, and investigating the performance of MPTCP with multi-RAT is of paramount importance.

However, studying the performance of MPTCP in a controlled cellular environment requires real testbed deployment of 4G long-term evolution (LTE)/5G systems, which incur high costs. In general, real hardware equipment is challenging to modify, upgrade, and scale, and it requires storage space. Therefore, many researchers tend to study the performance of MPTCP using computer simulations.

Network Simulator version 3 (ns-3) is one of the most widely used simulators for cellular networks. It is an open-source discrete-event network simulator licensed under the GNU GPLv2 license [7]. Ns-3 offers multi-RAT simulation capabilities, with Wi-Fi, LTE (LTE-Advanced (LTE-A), License Assisted Access (LAA), LTE-Unlicensed (LTE-U)), and Wireless Gigabit (WiGig). LTE-EPC Network Simulator (LENA) is the LTE module of ns-3 developed by Centre Tecnològic de Telecomunicacions de Catalunya (CTTC) [8]. 5G-LENA is developed as an extension to LENA module [9]. However, MPTCP is still not available as a module in any stable version of ns-3. Alternatively, the Direct Code Execution (DCE) feature of ns3 enables the use of the Linux kernel implementation of MPTCP. DCE is a module of ns-3 that allows the execution of existing implementations of userspace and kernel space network protocols/applications within ns-3 [10].

The objective of this research is to assess the MPTCP simulation fidelity of ns3-DCE. We test the credibility of MPTCP simulation by comparing its performance with the experimental outcomes of a real hardware testbed replicating the exact simulation scenarios. The testbed consists of a real LTE evolved packet core (EPC) with a real eNodeB, real Wi-Fi networks, and an Ethernet-based backbone network. Specifically, we investigate the simulation performance of four congestion control algorithms when used by MPTCP in a multi-RAT environment of LTE and Wi-Fi. The research focuses mainly on two performance criteria, namely, data transfer throughput and round trip time (RTT). Using similar network setups in the hardware testbed and the ns-3 simulator, we compare the MPTCP performance for Cubic, OLIA, LIA, and wVegas congestion control algorithms for different flow sizes. We consider two setups, one with a bottleneck link and another without a bottleneck link, to assess the performance of MPTCP simulations. A bottleneck link refers to the network communication link used by both the subflows of an MPTCP connection. The bottleneck existence affects how the congestion control algorithm works. If congestion is detected in one subflow, this affects the amount of traffic pushed on the other by the congestion control algorithm, especially if they share the same link capacity. Although the design goals of MPTCP congestion control includes improved throughput, friendliness (not to harm other flows), and load balancing (balancing congestion), not all of them are satisfied by all the algorithms. For instance, LIA is normally able to achieve fairness and optimal balancing of congestion between subflows simultaneously. On the other hand, OLIA satisfies both load balancing and responsiveness to varying network conditions. However, wVegas is more responsive when compared to LIA and OLIA. For this reason, we study the performance of different algorithms using the two mentioned setups.

This paper makes the following contributions. First, we analyze whether the uplink MPTCP throughput results obtained by the ns-3 simulator match the testbed throughput results. We analyze the difference in throughput performance of the aforementioned congestion control algorithms between simulation and hardware experiments for the two different setups. Second, the downlink MPTCP throughput achieved by simulation and hardware testbed is investigated. This also allows testing the fidelity of the LTE module of ns-3. Furthermore, we comparatively explore the impact of the flow lifetime on the throughput performance obtained by the simulator and testbed for the uplink and downlink. Third, the RTT performance of individual subflows is analyzed in different setups to estimate how faithful the MPTCP implementation of the simulator is with respect to the real hardware implementation. Fourth, the MPTCP throughput performance credibility is evaluated in ns-3 during the LTE handover.

The rest of the paper is organized as follows. Section 2 introduces the most relevant research works in the literature. Section 3.4 provides a basic background of Cubic, LIA, OLIA, and wVegas algorithms. The experimental setups, including the used hardware and software tools, are described in Section 4.2. Section 5 presents the details of the simulation model. The testbed and simulation results are discussed in Section 6. Finally, Section 7 concludes this work.

## 2. Related Works

The ns-3 simulator [7] was introduced in 2008 as a successor for the ns-2 simulator. However, ns-3 is not backward compatible with ns-2. Ns-3 is entirely written using C++ and supports python scripting, whereas ns-2 is partly written using C++ and partly using TCL scripting language. One of the major advantages of ns-3 over ns-2 is that the ns-3 supports the direct code execution feature, which allows the simulator to use real applications without code change. Moreover, ns-3 has been designed to be easily integrated into real testbeds and virtual machine environments [11]. The ns-3 simulator has been gaining popularity among researchers since its advent. Currently, it is widely adopted, as documented in [12]. A significant number of MPTCP research works depend on the ns-3 simulator. In the research articles [13,14,15,16], the authors propose implementations of MPTCP in ns-3. Some of these MPTCP implementations have been used to study MPTCP performance as in the sequel. The authors of [17] evaluate the impact of various packet reordering solutions on the throughput performance of MPTCP over different congestion control algorithms using ns-3 simulations. Li et al. propose a new congestion control approach to improve the MPTCP performance in data center networks. They utilize the ns-3 simulator to evaluate the goodput performance of MPTCP with the proposed congestion control method. In order to improve the performance of short flows in MPTCP, the authors of [18] propose a flow-based transmission optimization algorithm for MPTCP. They validate the proposed algorithm using ns-3 computer simulations. The authors of [19] analyze MPTCP in a cooperative relay-based LTE network and propose some extensions to MPTCP to guarantee a stable throughput. They validate the proposed model using the ns-3 simulator (LTE/Wi-Fi ns-3 models and implementation of MPTCP is used).

Since the addition of DCE functionality in ns-3 [10], some researchers have been able to exploit the Linux kernel implementation of MPTCP in ns-3 [20]. The authors of [21] utilize ns-3 DCE (Linux MPTCP) to analyze the effect of path characteristics (RTT and bandwidth) and packet scheduling policies on the throughput performance of MPTCP. Hashimoto et al. evaluate the throughput performance of MPTCP under varying factors such as bandwidth, delay, and frame error rate using ns-3 DCE. An analytical model to calculate the MPTCP throughput and latency is proposed and validated using ns-3 DCE in [22].

Other researchers leverage the LENA module (LTE) [8] and the Wi-Fi modules [23] of ns-3 to build a multipath heterogeneous wireless network environment. In [24], the authors analyze the throughput performance of MPTCP in a heterogeneous wireless network of LTE and Wi-Fi, where a Wi-Fi subflow experiences multiple handoffs. They propose congestion control strategies suitable for the handoff scenario and validate the proposed method using ns-3 DCE. The authors of [25] employ ns-3 DCE to evaluate their proposed software-defined networking (SDN) approach to improve fairness among single-path flows and MPTCP multipath flows (LTE and Wi-Fi). An MPTCP-based mobility support scheme for LTE networks is developed in [26]. The authors claim that the proposed solution enables seamless traffic continuity after a handover with serving/packet gateway relocation and validate this using ns-3 DCE. Gao et al. [27] propose a scheduler for MPTCP in SDN. They claim that the proposed scheduler improves throughput performance and minimizes the cost for users, according to the conducted ns-3 DCE simulations.

As MPTCP is expected to play a significant role in the 5G networks, researchers have started to focus on MPTCP with mmWave networks. Polese et al. provide a performance evaluation of single-path TCP and MPTCP with mmWave links using ns-3 DCE [28]. Recently, another multipath-based protocol named multipath QUIC was introduced [29], but to the best of the authors’ knowledge, no ns-3 implementation is available yet for this protocol.

Apparently, the discussed research works indicate a strong reliance on the ns-3 simulator. However, to the best of our knowledge, there is no other research work in the literature that examines the fidelity of MPTCP simulations using ns-3 DCE. This paper investigates the fidelity of ns-3 DCE by comparing the MPTCP performance results obtained by the ns-3 simulator and a real hardware testbed in two different real-world connectivity scenarios. Our study focuses on Cubic, LIA, OLIA, and wVegas algorithms and considers both long- and short-lived data flows.

## 3. Overview of MPTCP Congestion Control Algorithms

The multipath congestion control algorithms such as LIA, OLIA, and wVegas aim at the fair and efficient distribution of network resources among the competing flows. The multipath congestion control is mostly applied at the congestion avoidance phase, whereas the other phases (slow start, fast retransmit, and fast recovery) remain the same as the standard TCP algorithm. Cubic is a TCP congestion control algorithm that runs separately on individual subflows when used with MPTCP. The following is a brief description of these algorithms.

### 3.1. Cubic

Cubic [30] has been the default congestion control algorithm in Linux since 2006. Recently, Microsoft also adopted Cubic as the default congestion control algorithm in Windows [31]. This algorithm uses the following cubic window growth function, independent of RTT, to achieve better performance in long fat networks.

(1) $$Wnd\left(T\right)=c{\left(T-t\right)}^{3}+Wnd_{max}$$

where *c* is a Cubic parameter, *T* is the elapsed time from the last window reduction, and *t* is the time period the above function takes to increase to $Wnd_{max}$, when there is no further loss event.

Note that Wnd(T), the window growth function, is executed upon receiving an acknowledgment (ACK) during the congestion avoidance phase. The cubic parameter, *c*, determines the aggressiveness of the Cubic algorithm in competing for bandwidth with other congestion control algorithms. According to RFC 8312 [30], the value of *c* should be set to 0.4 to achieve a good balance between TCP-friendliness and window increase aggressiveness.

Cubic operates in three modes (TCP friendly, Concave, and Convex) depending on the current congestion window (cwnd). It operates in a TCP-friendly region when the cwnd is small to ensure that Cubic is not penalized relative to standard TCP. When the cwnd is less than $Wnd_{max}$, the window grows more slowly (concave region), whereas the window increases quickly (convex region) when the cwnd is greater than $Wnd_{max}$.

### 3.2. LIA

In the LIA algorithm [32], when an ACK is received for a subflow *r*, in the congestion avoidance phase, the congestion window grows by the following equation.

(2) $$min(\beta B\frac{MSS_{r}}{Wnd_{sum}},B\frac{MSS_{r}}{Wnd_{r}})$$

where *B* is the number of the acknowledged bytes, $MSS_{r}$ is the maximum segment size of subflow *r*, $Wnd_{r}$ is the congestion window of subflow *r*, $Wnd_{sum}$ is the sum of the congestion window of all subflows, and β is the aggressiveness parameter of the subflow given by

(3) $$\beta =Wnd_{sum}\frac{max(\frac{Wnd_{r}}{RTT_{r}^{2}})}{\sum_{r=1}^{n}\frac{Wnd_{r}}{RTT_{r}^{2}}}$$

where *n* is the number of subflows and $RTT_{r}$ is the round trip time of the subflow *r*. LIA meets the goals of improved throughput and friendliness. However, in order to achieve responsiveness, LIA departs from optimal load balancing.

### 3.3. OLIA

OLIA [33] was introduced as an alternative to LIA to provide both load balancing and responsiveness. The following congestion window increase function of OLIA is called when a subflow *r* receives an ACK.

(4) $$\left(\frac{\frac{Wnd_{r}}{RTT_{r}^{2}}}{{\left(\sum_{r=1}^{n}\frac{Wnd_{r}}{RTT_{r}^{2}}\right)}^{2}}+\frac{\beta_{r}}{Wnd_{r}}\right)\;B\;MSS_{r}$$

The congestion window is increased by summing up two terms. The first term is expected to guarantee optimal load balancing, whereas the second term is anticipated to preserve the responsiveness characteristic. The $\beta_{r}$ parameter in the second term is specific to each subflow *r*, and it is computed based on the quality of paths according to

$$\beta_{r}>0,\;If\;r\in Collected_{paths}\;\beta_{r}<0,\;If\;r\in Max\_win_{paths}\;\beta_{r}=0,\forall \;r,\;If\;all\;paths\;\in \;Best_{paths}\;have\;a\;maximum\;congestion\;window$$

where $Best_{paths}$ is a set of paths that are best for MPTCP connection (have maximum $l_{r}^{2}/RTT_{r}$); $l_{r}$ is computed based on the number of bytes transmitted between last two losses), $Max\_win_{paths}$ is a set of paths with a maximum congestion window, and $Collected_{paths}$ is a set of paths in $Best_{paths}$ but not in $Max\_win_{paths}$.

### 3.4. wVegas

wVegas [34] is a delay-based congestion control algorithm originated from TCP-vegas. Unlike LIA and OLIA, wVegas uses packet queueing delay as a sign of congestion. On each transmission round, the congestion window of subflow *i* ($W_{i}$) is updated in two steps. In step 1, the $W_{i}$ is incremented or decremented based on the parameters $\alpha_{i}$ and $diff_{i}$, as in the following:

If $diff_{i}$ > $\alpha_{i}$,

(5) $$W_{i}=W_{i}-1$$

If $diff_{i}$ < $\alpha_{i}$,

(6) $$W_{i}=W_{i}+1$$

According to (7), $diff_{i}$ is calculated as the difference between expected sending rate and actual sending rate. α_i is adjusted whenever it is smaller than $diff_{i}$ based on the ratio between current sending rate of subflow *i* and total rate of all the subflows as in (8).

(7) $$diff_{i}=\left(W_{i}/base\_RTT_{i}-W_{i}/RTTi\right)\;base\_RTT_{i}$$

(8) $$\alpha_{i}=\left(\left(W_{i}/base\_RTT_{i}\right)/total\_rate\_of\_all\_i\right)\;\alpha_{total}$$

In Step 2, the link queue variation is computed as

(9) $$q_{i}=RTT_{i}-base\_RTT_{i}$$

where $RTT_{i}$ is the average observed RTT and $base\_RTT_{i}$ is the minimum measured RTT of subflow *i*. When the current link queue variation is greater than 2q_i, the W_i is decreased using

(10) $$W_{i}=\;W_{i}\;\left(base\_RTT_{i}/2RTT_{i}\right).$$

wVegas algorithm is expected to be more responsive to the network congestion changes when compared to the other algorithms. It can achieve faster convergence with timely traffic shifting during network congestion.

In summary, LIA, OLIA, and wVegas are designed with multipath in mind. They consider all the multipath subflows in an MPTCP connection to calculate the congestion window growth function of each subflow. The calculation of the congestion window growth function in LIA depends mainly on the RTT value. OLIA takes both the RTT and the last loss event into account to determine the appropriate increase in the congestion window. Based on TCP Vegas, wVegas identifies network congestion in an early stage (before a loss happens) by observing the minimum RTT value of each subflow using the queuing delay and then gives each flow a weight based on the congestion of its path. Alternately, Cubic is designed to be a single-path congestion control algorithm. Therefore, it increases the congestion window of a subflow based mainly on its loss events without taking the status of the other subflows into account.

## 4. Testbed Description

In this section, we describe the testbed and provide the details of the configurations and tools utilized.

### 4.1. Experimental Setups

The hardware testbed is built using 10 dual-homed wireless nodes (five sender–receiver pairs) installed with Linux kernel implementation of MPTCP (version-0.89). The nodes concurrently send data packets using two real wireless networks; one is a 4G/LTE network and the other is a Wi-Fi network. The dual-homed wireless nodes access the wireless medium based on the technology used by each network. In Wi-Fi networks, the access to the medium is done using the IEEE 802.11 distributed coordination function (DCF), which follows the carrier-sense multiple access with collision avoidance (CSMA/CA) mechanism, as described in the IEEE 802.11 standard [35]. This mechanism is based on a random backoff procedure that is triggered when the channel changes its state from busy to idle. In this procedure, a backoff timer is randomly chosen from a contention window, which is doubled after each unsuccessful transmission. The LTE network follows the access mechanism defined by the 3GPP standard Release 8 [5]. It uses single-carrier frequency division multiple access (SC-FDMA) to access the uplink channel, whereas it uses orthogonal frequency division multiple access (OFDMA) to access the downlink one. Each network technology uses a different frequency band—5 GHz for Wi-Fi and 2.6 GHz for LTE.

**Figure 1:**
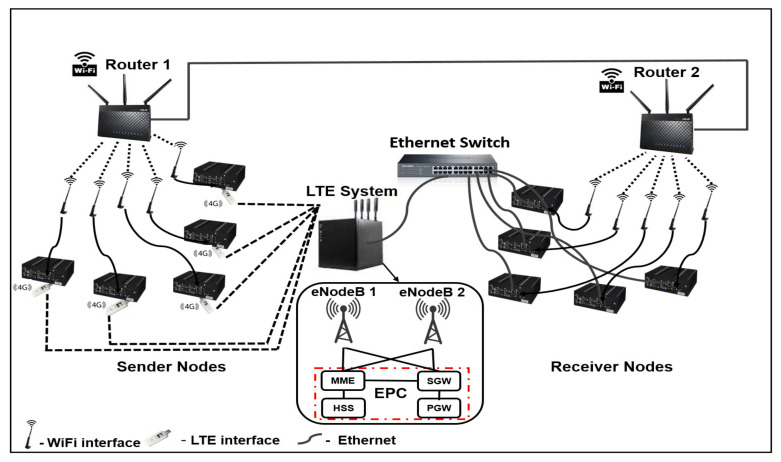
Testbed Setup 1.

We consider two topologies in this research: a setup without a bottleneck link (Setup 1) and a setup with such as a link (Setup 2). Figure 1 shows the architecture of Setup 1, which contains dual-homed wireless sender nodes. Each node hosts two wireless network interfaces. One interface is connected to a Wi-Fi network via Router 1, and the other is connected to the LTE network via a real eNodeB. However, in the case of the receiving nodes, one interface is a wired interface and the other is a wireless interface. The wired interface is connected to the LTE backbone network via an Ethernet switch. The wireless interface of the receiving nodes is connected to another Wi-Fi network via Router 2, which uses a different radio channel than the Wi-Fi network of the sending nodes. The LTE backbone network consists of an evolved packet core (EPC), which includes the mobility management entity (MME), serving gateway (SGW), and packet data network gateway (PGW). The configuration of Setup 1 makes the subflows of the same MPTCP connection independent of each other (no common bottleneck link between them).

**Figure 2:**
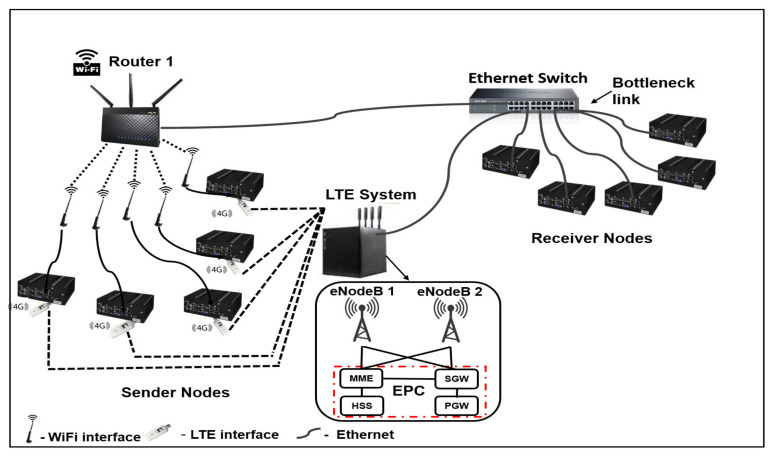
Testbed Setup 2.

Figure 2 reveals a scenario where the subflows of one MPTCP connection share a bottleneck link. The interfaces of the dual-homed sender nodes in Setup 2 are connected the same way as in Setup 1. However, the receiver nodes connect to both the LTE backbone network and the Wi-Fi network provided by Router 1 via an Ethernet switch. As revealed in Figure 1 and Figure 2, the testbed includes two real eNodeBs to realize S1 LTE handover.

### 4.2. Hardware and Software Tools

The dual-homed wireless nodes are single-board computers installed with the server version of Ubuntu Linux. The boards are equipped with an Intel Atom Z530 processor running at 1.6 GHz clock speed with 1 GB of memory. The Linux kernel implementation of MPTCP (version 0.89) provides flexibility for the nodes to select the congestion control algorithm, path manager, and packet scheduler to be used. It uses the standard MPTCP packet structure, as described by [36]. This kernel implementation is available for PC-based (x86) Linux distributions, such as Ubuntu, but not suitable to run, as it is, over Android. Thus, we use Ubuntu as a widespread Linux distribution in the testbed and also to run ns-3 DCE-based simulation with exactly the same kernel for a fair comparison. We use *fullmesh* path manager and the *default* RTT-based packet scheduler in our experiments. For all the available congestion control algorithms (Cubic, LIA, OLIA, and wVegas), the nodes are installed with several software programs to perform the experiments. Data traffic is generated using the *iperf* tool, which allows the adjustment of TCP-related parameters like receiver buffer size and data volume size. Tcpdump and Wireshark packet capturing tools are used to capture and analyze the generated data traffic. The data transfer commands are issued simultaneously to all the sending nodes using the parallel secure shell (PSSH) software tool. The dual-homed wireless nodes are time synchronized using the Network Internet Protocol (NTP). Each node runs an NTP client that synchronizes to a local NTP server. Time synchronization is performed for each experiment run.

The MPTCP-enabled nodes host two USB network adapters. The first is a Huawei LTE adapter to connect to the real LTE network [37]. These LTE adapters follow LTE Release 8 [5] that allows an uplink speed of 50 Mbps and a downlink speed of 150 Mbps. The LTE network includes two cells with two real eNodeBs that are connected to the EPC. The eNodeBs are software-defined radios operating in the FDD mode at 2.6 GHz (band 7). They are capable of supporting bandwidth up to 20 MHz. According to the standard LTE Release 14 [5], the EPC includes the home subscriber server (HSS), MME, SGW, and PGW.

Two Wi-Fi networks are constructed using two access points (wireless routers). The two networks are connected via an Ethernet switch. The second USB adapter of all nodes is a Wi-Fi adapter. The routers and the Wi-Fi adapters are dual-band and capable of supporting IEEE 802.11a/g/n/ac. Indeed, the relatively modern Wi-Fi standards, such as IEEE 802.11n/ac, use advanced techniques such as transmit diversity and channel aggregation [35]. IEEE 802.11ac also has additional capabilities such as working with a wider bandwidth and higher-order modulation schemes [38]. This leads to increasing the possible achievable rate of the Wi-Fi interface, which reduces the RTT, and hence affects the calculation of the congestion window size of most of the MPTCP congestion control algorithms. Nevertheless, the routers are configured as IEEE 802.11a access points because the rate adaptation algorithms do not work appropriately with 802.11n/ac implementation of ns-3 version 3.25 [39]. This issue is fixed only starting from version 3.26 [40]. However, the used DCE release (1.8), which contains the MPTCP kernel implementation, is supported up to version 3.25 only.

## 5. Simulation Model

This section shows how the exact replication of the hardware testbed is built using the ns-3 simulator. It also includes the details of the ns-3 simulator model and utilized parameters.

### 5.1. Simulation Setups

This research uses the ns-3 version 3.25 with DCE 1.8. The DCE tool allows utilizing the Linux networking stack within the ns-3 simulator. Generally, the network protocols in an early development phase are first implemented in the Linux kernel. Testing such new protocols using their Linux kernel implementation is more realistic than implementing them using a simulator.

As mentioned in Section 2, some researchers have proposed different implementations of MPTCP in ns-3. However, none of them are integrated with the ns-3 release, as they are not fully compliant with the MPTCP specifications. Alternately, the DCE framework provides the flexibility of using the Linux kernel implementation of MPTCP within ns-3. Therefore, in this research, we use the Linux kernel MPTCP implementation provided by the MPTCP protocol designers through the DCE framework. We use in the simulator the same version of MPTCP (0.89) employed in the hardware testbed. The MPTCP parameters such as path manager and packet scheduler are configured with the same values as the testbed. Likewise, ns-3 DCE also gives the flexibility of selecting the congestion control algorithm (Cubic/LIA/OLIA/wVegas) to be used. The ns-3 DCE also provides the benefit of utilizing POSIX-socket based applications. This allows using the traffic generation tool *iperf* the same as in the testbed. Thus, the DCE feature offers an emulation mode that allows the simulated experiments to run in real time, similar to the hardware testbed.

**Figure 3:**
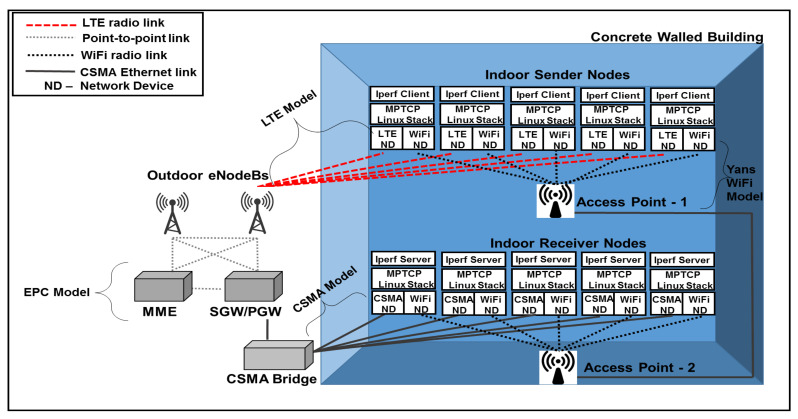
Simulation Setup 1.

Figure 3 shows the replication of testbed Setup 1 in the ns-3 simulator. The simulated nodes are installed with MPTCP Linux stack instead of the default ns-3 stack. Like the testbed, the sending nodes are equipped with LTE and Wi-Fi network interfaces (“network devices” in ns-3 terms). The LTE network device is connected to the LTE network by attaching to an eNodeB, whereas the Wi-Fi network device is connected to the Wi-Fi network via Access Point-1. One interface of the receiving nodes is also connected to a Wi-Fi network via Access Point-2. A wired CSMA link is created between the PGW of the EPC and the receiving nodes to replicate the Ethernet switch as in Setup 1 of the testbed.

**Figure 4:**
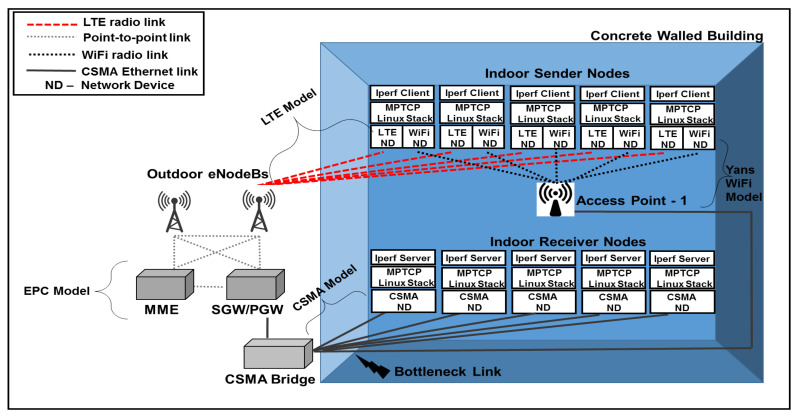
Simulation Setup 2.

Setup 2 of the testbed, where a bottleneck link exists, is recreated in the simulator, as shown in Figure 4. The configuration of the sending network and the connectivity of the network nodes are the same as in Setup 1. The bottleneck link leading to the receiving network is created using a bridge (similar to the Ethernet switch in the testbed) that connects the receiving nodes to the LTE network via the PGW and the Wi-Fi network via AP-1. As revealed by Figure 3 and Figure 4, the LTE network includes two eNodeBs. Since the S1-based handover is still not implemented in ns-3, we only simulate the X2-based handover between the eNodeBs.

### 5.2. Simulator Parameters and Configuration

Every node in the simulator has two network devices (same as network interface cards in real nodes). The simulator is configured to use a constant-position mobility model. Thus, the nodes remain stationary during the data transfer.

We simulate the LTE network used in the testbed by utilizing LENA, an LTE/EPC simulation platform of ns-3. The core network of LTE is simulated using the ns-3 EPC model, which includes one MME and one more entity that assumes the SGW and PGW’s responsibilities. The interfaces between these entities are implemented as high-speed point-to-point links to mimic the hardware implementation. The LTE model of ns-3 implements the LTE radio protocol stack at the eNodeB and the communicating nodes (UEs). The simulator’s LTE stack includes all the layers of the real LTE stack, such as Radio Resource Control (RRC), Packet Data Convergence Protocol (PDCP), Radio Link Control (RLC), Medium Acess Control (MAC), and Physical (PHY). The LTE stack parameters are configured, as listed in Table 1, to replicate the LTE settings of the testbed. Thus, the essential parameters such as the MAC scheduler, sounding reference signal (SRS) periodicity, maximum modulation coding scheme (MCS), and maximum transmission unit are set to the same value as in the configuration of the LTE network of the hardware testbed. The number of resource blocks is set to 100 to achieve a bandwidth of 20 MHz as in the testbed. The eNodeBs use the FDD mode in band 7 with a downlink frequency of 2660 MHz and an uplink frequency of 2540 MHz.

The path loss model, transmission power, and eNodeB/UEs’ positions are adjusted so that the average MCS value reported by the UEs in the simulator matches the one reported by the LTE hardware. As revealed in Figure 3 and Figure 4, the LTE UEs (sender nodes) are located in an indoor environment (inside a building), whereas the eNodeB is located outside this building. This setting allows applying the HybridBuildingPropagation loss model of ns-3, which provides suitable flexibility to adjust its parameters to obtain similar path loss to the testbed’s LTE network since it includes a path loss model with distance-dependent slope and path loss elements (external/internal wall loss and height loss). The indoor–outdoor scenario for the UEs and the eNodeB in the HybridBuildingPropagation model is based on the ItuR1411 path loss model [41]. In this model, the coordinates of the UEs and eNodeB determine whether the path is Line of Sight (LOS) or Non-LOS so the loss can be calculated accordingly. The considered simulation setup only involves LOS paths. According to the ItuR1411 path loss model, the path loss for LOS paths is characterized by two slopes and a single breakpoint. The path loss at the breakpoint is given by

(11) $$L_{bp}=20\times log_{10}\left(\lambda^{2}/\left(8\times \pi \times h1\times h2\right)\right)$$

where λ is the wavelength, h1 is height of the node, and h2 is the height of the eNodeB. The breakpoint distance is defined as

(12) $$R_{bp}=\left(4\times h1\times h2\right)/\lambda$$

The overall loss is given by

(13) $$Loss_{I1411}=\left(Loss_{low}+Loss_{up}\right)/2$$

where the loss upper bound ($Loss_{up}$) and loss lower bound ($Loss_{low}$) can be calculated

If *d* <= $R_{bp}$, as

(14) $$Loss_{low}=L_{bp}+20\times log_{10}\left(dist/R_{bp}\right)$$

(15) $$Loss_{up}=L_{bp}+20+25\times log_{10}\left(dist/R_{bp}\right)$$

If *d* > $R_{bp}$, it can be obtained from

(16) $$Loss_{low}=L_{bp}+40\times log_{10}\left(dist/R_{bp}\right)$$

(17) $$Loss_{up}=L_{bp}+20+40\times log_{10}\left(dist/R_{bp}\right)$$

The parameter *d* refers to the distance between the node and the enodeB. The total loss incurred by the hybrid building propagation loss model is given by

(18) $$Loss_{total}=Loss_{I1411}+EWL+HL$$

The external wall loss (EWL) in a concrete-walled building with windows is set to a constant value of 7 dBm in ns-3. Alternately, the height loss (HL) is zero as a single-floor building is considered.

The Wi-Fi network of the testbed is simulated using the yet another network simulator (Yans) [42] Wi-Fi model of ns-3. The propagation loss is based on the log distance model given by

(19) $$L=L_{0}+10nlog\left(d/d_{0}\right)$$

where *L* is the path loss (dB), *n* is the path loss exponent, and *d* is the distance (m). The reference path loss $L_{0}$ is set to 46.6777 dB, and the reference distance $d_{0}$ is kept as 1 m.

Table 2 shows that the parameters such as frequency, rate manager, and channel width, among others, are configured with values similar to the testbed Wi-Fi configuration. For instance, by employing the AarfWifiManager, the Wi-Fi channel access rate is changed adaptively the same way as in the hardware testbed. The Wi-Fi power-related parameters such as TX/RX gain, TX power levels, RX noise figure, and RX sensitivity are adjusted in a way that makes the reported RSSI (receiver signal strength indicator) at the access point and nodes’ Wi-Fi interfaces in the simulator and testbed matching one another. This leads to a similar average channel access rate in the simulator and testbed.

## 6. Results

In this section, the simulation and hardware testbed results are presented, compared, and discussed. To achieve accurate statistics, all the experiments are repeated 40–50 times with the testbed and the simulator. The experimental results are represented as average values of the sample runs with error bars. The length of the error bar indicates the standard deviation around the mean. The absence of any overlap between the error bars indicate a statistically significant difference.

Our study focuses on the following performance aspects of the DCE implementation of the MPTCP protocol in the ns-3 simulator. First, we explore the uplink/downlink MPTCP achievable throughput with the aforementioned congestion control algorithms. After that, we study the MPTCP round trip time (RTT) values. Next, we evaluate the MPTCP performance during LTE handover.

In all the aspects mentioned above, the performance of MPTCP in the ns-3 simulator is compared with the hardware testbed. The MPTCP throughput is measured by the time required to transmit data between the sender and receiver applications. Throughout this data transfer, the RTT is measured by calculating the time taken to send packets and receive acknowledgments. We focus on two data (flow) sizes unless otherwise mentioned. One represents a relatively short flow (20 MB), whereas the other is a long-lived flow (75 MB). Using a larger data volume leads to no noticeable impact on the experiment outcomes except increasing the file transmission time, which is prohibitive to repeating each experiment for a sufficiently large number of samples in a reasonable time. The two flow sizes can emulate different traffic patterns [43] and their impact on throughput. For instance, short-lived flows emulate the transmission of a relatively small chunk of data followed by a relatively long period of no data transmission, whereas long-lived ones imitate transferring a large amount of data, such as transferring a large file or browsing a large website. During LTE handover, the MPTCP performance evaluation is conducted for the S1-based handover in the testbed and the X2-based handover in the simulator. Note that all results are obtained with similar configuration parameters of Wi-Fi and LTE networks in the simulator and testbed, as discussed in Section 4.2 and Section 5.

### 6.1. Uplink MPTCP Throughput

During uplink transmission, unlike real-time, when we try to start transmitting simultaneously on all nodes in the ns-3 simulator using DCE-*iperf*, the data transfer starts exactly at the same time on all nodes with no slight difference. Therefore, we compare the real hardware experimental results with the simulation case where all the sender nodes are scheduled to start their transmission nearly at the same time (10 ms second jitter is applied as recommended by ns-3 developers and experts [44,45]). This allows the nodes to compete for the available network resources similar to practical scenarios.

**Figure 5:**
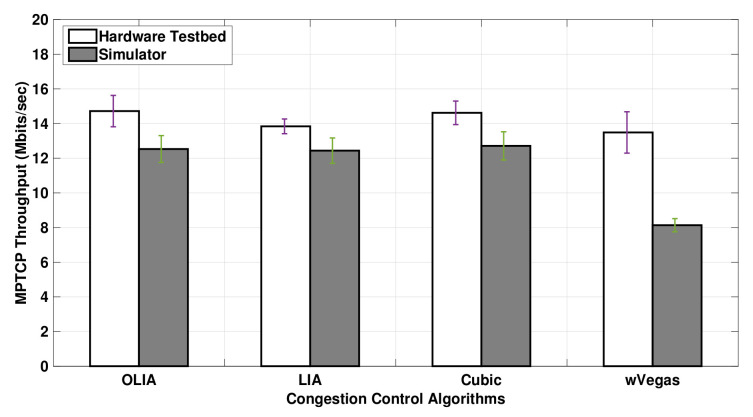
Setup 1 uplink MPTCP throughput for 20 MB flows.

Figure 5 shows the MPTCP throughput for OLIA, LIA, Cubic, and wVegas algorithms obtained with the real hardware testbed and ns3-simulations using Setup 1. In this experiment, the data volume transferred by the sending nodes is 20 MB. The results reveal that the throughput observed with the simulator is generally smaller than the testbed throughput results, where wVegas exhibits the largest and LIA the smallest throughput difference between the simulator and the testbed.

The simulation results show the same throughput performance among the algorithms except for wVegas. However, the testbed demonstrates different throughput performance with different congestion control algorithms.

**Figure 6:**
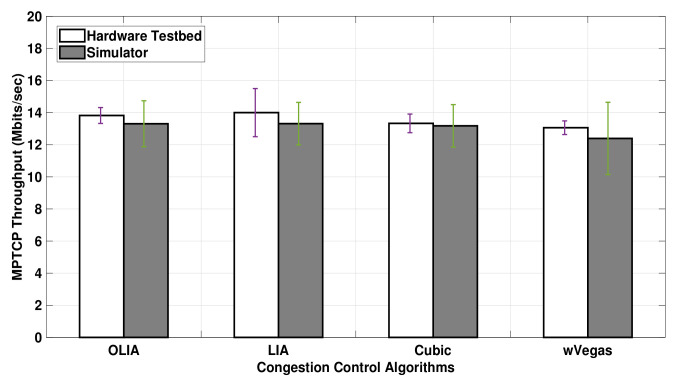
Setup 1 Uplink MPTCP Throughput for 75 MB flows.

Figure 6 presents the uplink MPTCP throughput obtained when the data volume is increased to 75MB. The figure shows that the MPTCP throughput achieved with long-lived flows in ns-3 is much closer to the testbed throughput results than short-lived flows. In particular, Cubic exhibits a close match between the simulator and the testbed results. Similar to relatively short-lived flows, the simulator, as shown in Figure 6, demonstrates almost no difference in the throughput performance among the congestion control algorithms for long-lived flows, except with wVegas. Alternatively, a slight difference in throughput is observed among the congestion control algorithms with the testbed.

**Figure 7:**
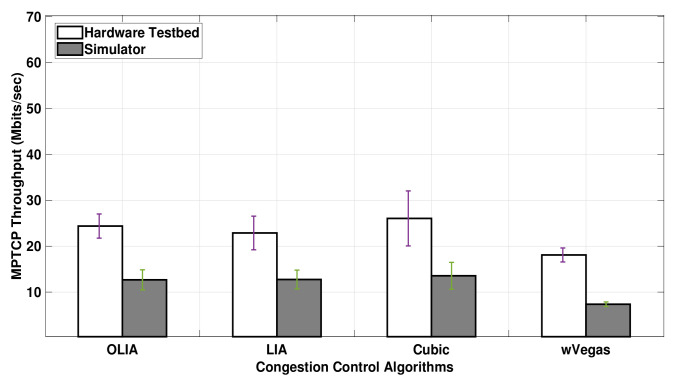
Setup 2 Uplink MPTCP Throughput for 20 MB flows.

For Setup 2, the testbed throughput results are also greater than the simulation results, as depicted in Figure 7. However, the throughput difference observed between the testbed and the simulator is significantly higher for 20 MB flows in Setup 2 than in Setup 1. Although the simulator shows the same throughput among the congestion control algorithms (except wVegas), the testbed shows a noticeable difference in throughput among them. Generally, the simulator gives different results from the hardware testbed for short-lived flows with Setup 2.

**Figure 8:**
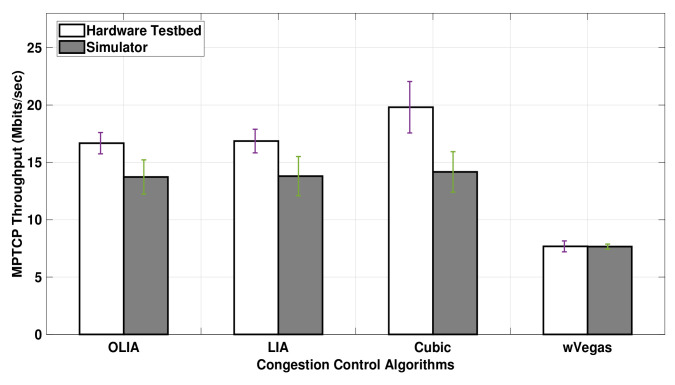
Setup 2 Uplink MPTCP Throughput for 75 MB flows.

The results from Figure 8 reveal that the difference between simulation and hardware testbed results in Setup 2 is smaller with long-lived flows (75 MB) than short-lived flows (shown in Figure 7). However, Figure 8 depicts that the throughput difference varies among algorithms since it is significant with the Cubic algorithm and almost vanishes with wVegas.

In short, the simulator generally underestimates the uplink MPTCP throughput performance with short-lived flows. However, the simulation results match the testbed ones for long-lived uplink flows when no bottleneck link exists. With long-lived uplink flows passing through a bottleneck link, the simulator results match the testbed results with wVegas, but it slightly underestimates the throughput of LIA and OLIA and gives a noticeable lower throughput than the testbed with Cubic.

### 6.2. Downlink MPTCP Throughput

**Figure 9:**
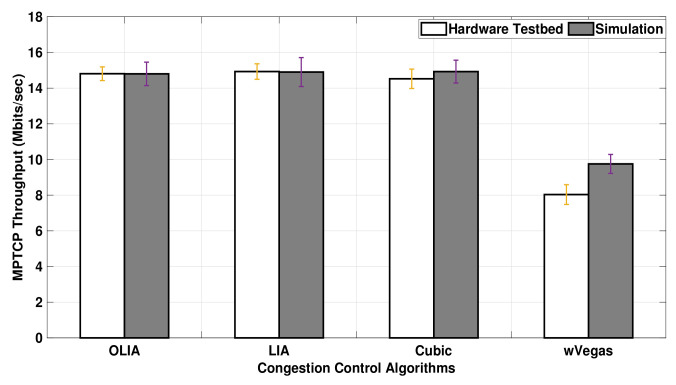
Setup 1 Downlink MPTCP Throughput.

The downlink experiments are performed by allowing the wireless nodes to download a 20 MB file from a single download server simultaneously over the LTE and Wi-Fi networks. Figure 9 depicts the downlink throughput performance of MPTCP in Setup 1. The results reveal that there is generally a match between the hardware and ns-3 simulator results, except for wVegas, where the simulator gives around 25% larger throughput compared with the testbed.

**Figure 10:**
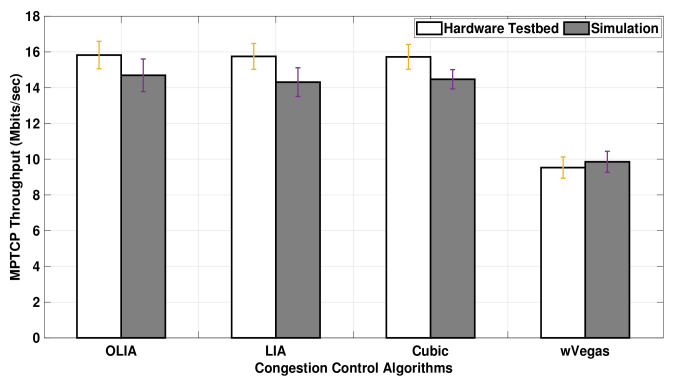
Setup 2 Downlink MPTCP Throughput.

Figure 10 reveals that the MPTCP downlink throughput of the testbed in Setup 2 is greater than the simulator (by around 10–15%) for all algorithms except wVegas, where there is a close match. However, both the simulator and the testbed show the same throughput performance trend among the studied congestion control algorithms.

In summary, the simulator throughput results match the testbed results when no bottleneck exists in the network configuration for all the studied algorithms except with wVegas, where the simulator results are higher than its testbed counterpart. On the contrary, wVegas throughput simulation results match the testbed ones when a bottleneck link exists, whereas the simulator slightly underestimates the throughput results of the rest of the algorithms.

### 6.3. MPTCP RTT

RTT is an influential parameter in the network performance of MPTCP. The default MPTCP scheduler makes path selection decisions based on the RTT of each available path. When multiple subflows are available to transmit data, the default scheduler sends the data segments to the subflow with the minimum RTT to fill its congestion window first. Multiple congestion algorithms directly rely on RTT to calculate the congestion window size such as LIA (3), OLIA (4), and wVegas (7) and (8).

**Figure 11:**
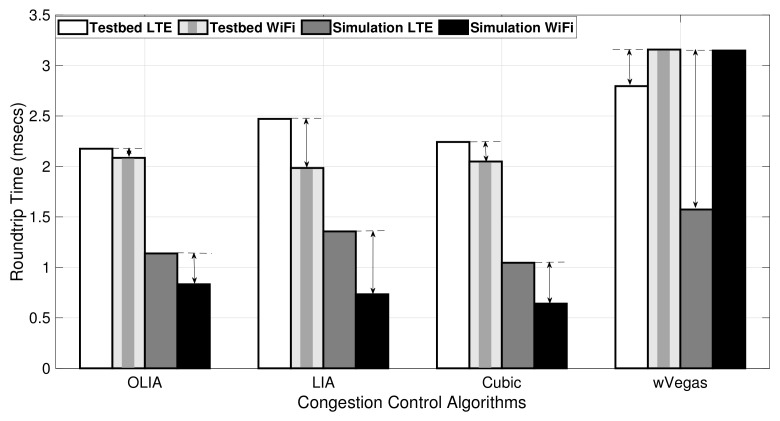
Setup 1 uplink MPTCP RTT for 20 MB flows.

Since the simulator MPTCP throughput results generally match the testbed results for downlink scenarios, we focus here on investigating the RTT for uplink experiments. Figure 11 shows the average RTT of LTE and Wi-Fi subflows observed during the uplink MPTCP experiment using Setup 1, whereas Table 3 presents the numerical RTT values and their corresponding standard deviation for the testbed (TB) and simulator (Sim). The figure depicts a clear difference in the RTT values, especially for the LTE interface, between the simulator and testbed, despite using the transmitter–receiver distance in the simulator that leads to recording the same LTE MCS and Wi-Fi signal at the receiver as the testbed following Equations (11)–(18) for the LTE network and Equation (19) (with Yans model) for the Wi-Fi network.

Indeed, the difference in the average RTT values between subflows has a direct impact on the performance of MPTCP. When there is a similar difference in the average RTT between the Wi-Fi and LTE subflows, a close match is noticed in the throughput performance between the simulator and the testbed results, as observed for the LIA algorithm in Figure 5. On the contrary, a significant average RTT difference is noticed with wVegas, which leads to a different throughput performance between the simulator and testbed. The reason behind the different behavior in wVegas is that it is sensitive to the measurement accuracy of the $base\_RTT_{i}$ parameter, which influences the calculation and evolution of the subflow weights ($\alpha_{i}$), and hence affects the effectiveness of the algorithm [34].

**Figure 12:**
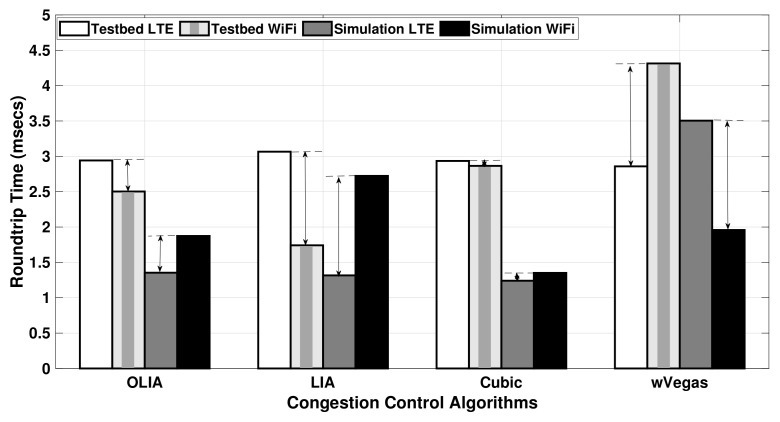
Setup 1 uplink MPTCP RTT for 75 MB flows.

Figure 12 shows the average RTT values, and Table 4 provides the corresponding numerical values with their standard deviation observed when transferring a large data flow (75 MB) over the uplink in Setup 1. As denoted by the difference arrow in Figure 12, the difference in RTT (measured between the Wi-Fi subflow and the LTE subflow in the testbed and the simulator) is close among all the congestion control algorithms. This translates to a small difference in the throughput results of the simulator and testbed for large data volume as revealed in Figure 6.

**Figure 13:**
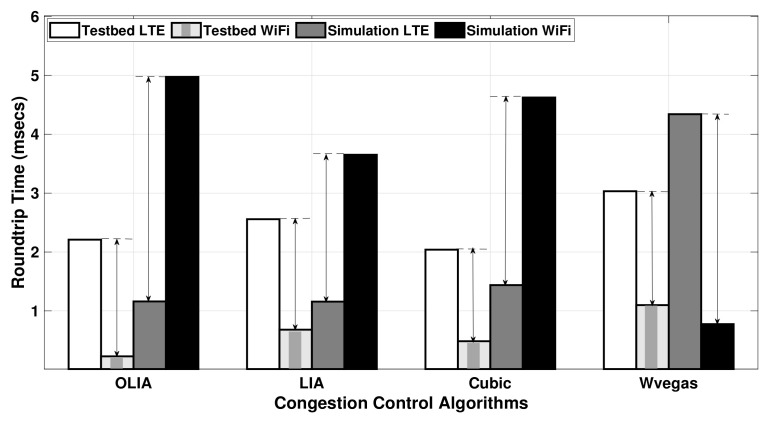
Setup 2 uplink MPTCP RTT for 20 MB flows.

Regarding the effect of bottleneck links, Figure 13 depicts the average RTT values observed in Setup 2 for a relatively small flow size (20 MB). In addition, Table 5 presents the average and standard deviation of the RTT values for the same experiment. Generally, a significant difference is noticed between the RTT values of the LTE subflow and the Wi-Fi subflow. However, it is clearly shown in the figure that the RTT difference of the subflows measured with the testbed is smaller when compared to the simulation RTT values. As a result, the simulator experienced a varied throughput performance compared with the testbed, as demonstrated in Figure 7.

**Figure 14:**
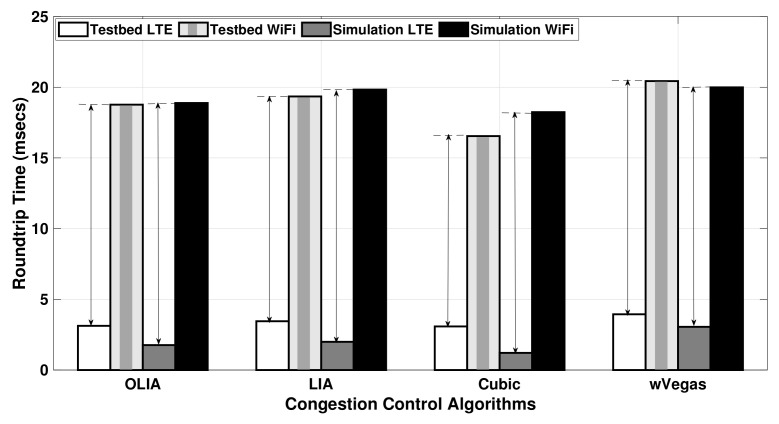
Setup 2 uplink MPTCP RTT for 75 MB flows.

For long-lived flows sharing a bottleneck link, Figure 14 displays the average RTT values observed with a larger data size (75 MB) in Setup 2, and Table 6 provides the standard deviation as well. The difference arrow apparently shows that the Wi-Fi subflow RTT value is 5–6 times greater than the LTE subflow RTT for all the congestion control algorithms for both the simulator and testbed. It is worth noting that when wVegas is used with the simulator, the RTT difference of the subflows measured is nearly similar to the testbed, as observed in Figure 14. This is the reason behind the close match observed in wVegas throughput performance in the simulator and testbed, as shown in Figure 8. The other algorithms, especially Cubic, demonstrate a relatively larger RTT difference between the LTE and Wi-Fi subflows when compared to the testbed.

In brief, the difference between subflows’ RTT values measured by the simulator and testbed with long-lived flows (75 MB) is significantly smaller than their counterparts with short flows (20 MB).

### 6.4. MPTCP Throughput with Handover

We study the impact on the performance of MPTCP when a handover is executed at the LTE system. Two types of handover procedures are investigated, namely X2-based handover and S1-based handover.

The X2-based handover is an intra-RAT handover, which depends on the interaction between the serving and target eNodeBs via the X2 interface. In this case, the handover decision is made by the serving eNodeB based on the measurement report received from the UE. The serving eNodeB then directly notifies the target eNodeB by sending a handover request message over the X2 interface. The core-network (MME/SGW) is involved only at the end of the X2-based handover procedure.

When there exists no X2 interface between the eNodeBs or when the handover is towards another RAT (inter-RAT handover), the S1-based handover procedure is utilized. In this procedure, the handover signaling happens through the MME—that is, when the serving eNodeB decides to perform a handover, it first notifies the MME via the S1 interface.

Both X2 and S1 handovers are hard handovers, which implies that a short service interruption is anticipated when the handover is performed.

The throughput performance of MPTCP handover is examined in the testbed by performing an S1-based handover while transmitting a 50 MB file from a sender node to a receiver node. The handover experiment is executed by gradually attenuating the transmission power level of the serving eNodeB while increasing the transmit power of the target eNodeB during the data transfer. The handover is initiated when the UE receives much less transmission power from the serving eNodeB than the target eNodeB. Thus, the LTE USB adapter of the sending node (UE) is forced to perform a handover to the target eNodeB.

An X2-based handover is investigated in the simulator since it is the only type of handover currently implemented in ns-3. This requires an X2 interface link to be created between two eNodeBs in the simulation scenario. Like the testbed, the handover is performed while transferring a 50 MB file from a sending node to a receiving node. The handover is triggered in the simulator while changing the relative position between the UE and the eNodeBs. Once the distance between the UE and the serving eNodeB becomes significantly longer than the distance between the UE and the target eNodeB, the handover occurs from the serving eNodeB to the target eNodeB.

The S1-based or X2-based handover is set to happen around the middle of the data transfer. The handover-related LTE parameters are kept the same in the testbed and the simulator. For instance, we use the same handover algorithm, A2A4-Reference Signal Received Quality (RSRQ), with the testbed and simulator. The Radio Link Control (RLC) layer’s transmission mode is set to Acknowledged Mode (AM) in both the testbed and simulator.

**Figure 15:**
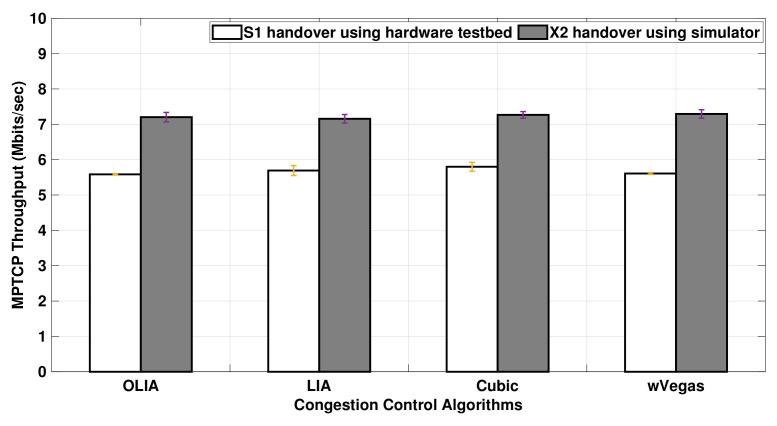
Setup 1 MPTCP throughput with handover for different congestion control algorithms.

Figure 15 shows the overall MPTCP throughput achieved when an LTE handover is performed in Setup 1. Higher throughput is observed with the simulator when compared to the testbed. However, the simulator is able to preserve the throughput trend observed among the congestion control algorithms in the testbed.

**Figure 16:**
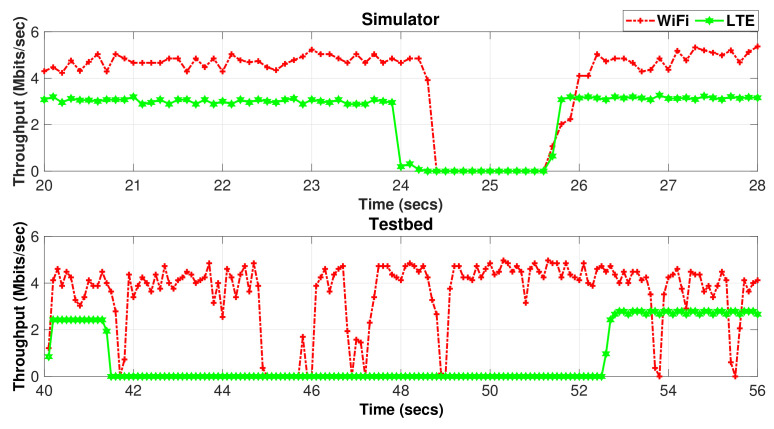
Setup 1 Wi-Fi/LTE subflow throughput variation with time during handover occurrence.

The difference between the simulator and test results can be explained by examining the instantaneous MPTCP throughput for Wi-Fi and LTE subflows measured by the simulator and testbed during the handover period, as revealed in Figure 16. The figure clearly shows that the hard handover results in an almost zero throughput of the LTE interface during the handover period. The throughput drop takes around 2 s during the X2-based handover in the simulator, whereas the S1-based handover takes around 10 s. This is an anticipated behavior since the S1-based handover involves exchanging signaling messages through the core network (MME/SGW), which, in turn, increases the duration of the handover procedure when compared to the X2-based handover.

**Figure 17:**
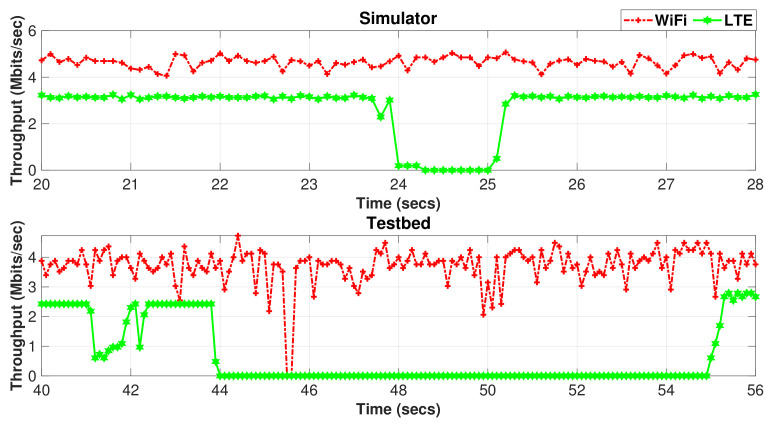
Setup 1 Wi-Fi/LTE subflow throughput variation with time for wVegas during handover occurrence.

A drop in the Wi-Fi throughput is also noticed during the X2-based handover for a shorter period than with LTE. In the testbed, the Wi-Fi throughput drops for a significantly shorter period than the simulator. This throughput behavior, as revealed in Figure 16, is observed with OLIA, LIA, and Cubic algorithms. It is attributed to the relatively slow update in the operation of the scheduler that tends to fill first the congestion window of the subflow with shorter RTT (LTE). However, the wVegas algorithm shows different performance with the Wi-Fi subflow during the handover period since the Wi-Fi throughput does not drop to zero or drops very briefly during the handover period, as shown in Figure 17, with the simulator and testbed, respectively. This is attributed to the fast ability of wVegas to adapt to network conditions.

**Figure 18:**
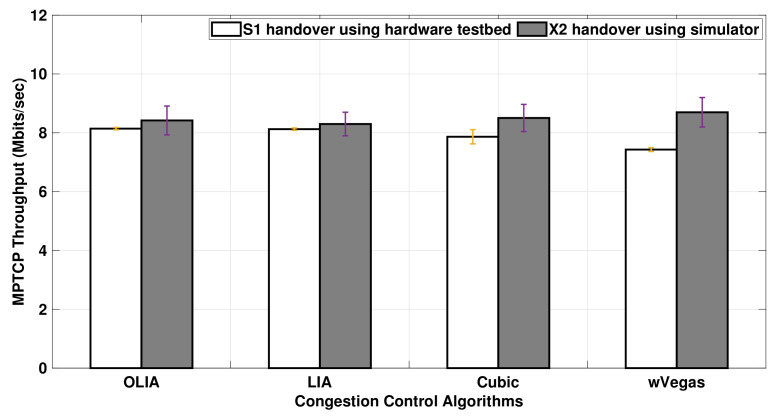
Setup 2 MPTCP throughput with handover for different congestion control algorithms.

The impact of the presence of a bottleneck link is studied in the handover experiments performed using Setup 2, as presented in Figure 18. The throughput observed in the simulator is in a close match with the testbed throughput results for OLIA, LIA, and Cubic. On the other hand, the throughput difference between the simulator and testbed becomes a bit more noticeable with wVegas.

In brief, with no bottleneck link, the MPTCP throughput simulation results are higher than the testbed results for all the studied algorithms when a handover scenario is performed during data transmission. However, the throughput results of the testbed match the simulator ones when the handover is performed in a network configuration with a bottleneck, except with wVegas, where the simulation throughput results are higher.

### 6.5. Discussion

From the presented results, it is evident that a significant statistical difference between the MPTCP throughput results of the ns-3 simulation and testbed is noticed in the following cases.

- In the case of uplink traffic, the simulator underestimates the MPTCP throughput with short-lived flows irrespective of the existence of a bottleneck link. However, with long-lived flows passing through a bottleneck link, the simulator slightly underestimates the throughput of LIA and OLIA, but it matches wVegas, and gives a noticeable lower throughput than the testbed with Cubic.
- In the case of downlink traffic, the simulator overestimates wVegas throughput when no bottleneck link exists. However, a slight underestimation of the throughput with all algorithms, except wVegas, is noticed when a bottleneck link is present.
- In the case of LTE handover, the simulator overestimates throughput when no bottleneck link exists. However, only the throughput of wVegas is overestimated when a bottleneck is present.

The main cause of the discrepancy observed with uplink and downlink traffic, as indicated in Section 6.3, is the different RTT values measured by the simulator compared with the RTT values obtained by the testbed. This is attributed to the fact that the ns-3 simulator does not implement the full control plane of the LTE stack, but a simplified one [46], which implies that it does not truly represent the delays caused by the control plane procedures. Indeed, having a different RTT over one network interface impacts the RTT of the other interface as the MPTCP scheduler first fills the congestion window of the interface with the lowest RTT. Moreover, the congestion window calculations of each subflow depend on the other for LIA, OLIA, and wVegas algorithms.

Moreover, the bottleneck presence impacts the RTT. This affects the difference that already exists between its values in the simulator and testbed. In addition, the performance of wVegas is highly affected by the accuracy of the RTT values [34], which affect the minimum measured RTT ($base\_RTT_{i}$) parameter, and hence influences the calculation and evolution of the subflow weights ($\alpha_{i}$). For most of the studied algorithms, ns-3 simulation results tend to better approach the testbed results for long-lived flows as the difference in RTT between the subflows (i.e., Wi-Fi and LTE) of an MPTCP connection measured in the testbed becomes closer to its simulator counterpart when averaged over a long time scale of data transmission.

The reason behind the discrepancy with LTE handover is because the ns-3 simulator implements only the X2-based handover, which is a shorter procedure compared with the S1-based handover. The rates of the different subflows of the same MPTCP connection often take a shorter time to stabilize (after an S1-based handover disturbance) when they share a bottleneck link as they directly affect one another, which leads to a less impact on the overall throughput.

## 7. Conclusions

The paper studies the fidelity of ns-3 simulations for dual-homed wireless nodes using Linux-based MPTCP kernel implementation embedded in the ns-3 DCE module. The study is hinged on comparing the ns-3 DCE simulation results with a testbed that allows several dual-homed (LTE and WiF) wireless nodes to communicate to one another via real Wi-Fi and LTE networks in a controlled environment using different MPTCP congestion control algorithms (LIA, OLIA, Cubia, and wVegas).

The investigated scenarios reveal apparent differences between the simulator and testbed throughput performance. Generally, it is observed that the data volume, the existence of a bottleneck link, and the employed congestion control algorithm impact the fidelity of the simulation results.

For instance, in uplink MPTCP packet transfer, the throughput results of the ns-3 simulator are not in a close match with the testbed results with short-lived flows. However, ns-3 DCE generally replicates the testbed throughput results to a good extent for long-lived flows except when the Cubic algorithm is used in a network configuration with a bottleneck link.

It is found that the MPTCP downlink throughput obtained by ns-3 DCE reasonably matches the throughput measured by the testbed when no bottleneck exists, except for wVegas. However, it slightly underestimates the MPTCP throughput for most of the studied algorithms with the presence of a bottleneck link in the network configuration.

Moreover, the ns-3 DCE overestimates the MPTCP throughput during handover compared with the testbed when no bottleneck link exists in the simulated network since it does not implement the S1-based handover as the testbed. However, the simulator’s MPTCP throughput results reasonably match the testbed results during the handover with the existence of a bottleneck for most of the studied congestion control algorithms as the subflow rates tend to stabilize faster after the handover disturbance.

Furthermore, it is found that the measured RTT values of the simulator and testbed for Wi-Fi and LTE subflows well justify the mentioned throughput performance. The main reason of the discrepancy between the simulator and testbed results lies in that the ns-3 DCE RTT values significantly differ from their testbed counterparts, although the signal level and MCS values are kept the same for both. This can be attributed to the simplified control plane of the simulator LTE implementation.

We believe that the ns-3 simulator can replicate the MPTCP throughput results of multihomed (Wi-Fi and LTE) nodes of a real testbed to a good extent only for long-lived uplink connections using LIA, OLIA, or wVegas algorithm and downlink connections using LIA, OLIA, or Cubic algorithm. This also applies to handover scenarios if S1-based handover is implemented.

## Figures and Tables

**Table 1:** LTE simulation parameters.

LTE Parameter	Value
LTE Access Mode	FDD
Number of Resource Blocks	100
DL-EARFCN	3150
LTE Scheduler	PfFfMacscheduler
SRS Periodicity	320 ms
eNodeB Tx-Power	30 dBm
eNodeB Noise Figure	5 dB
Maximum Uplink MCS	23
Maximum Downlink MCS	28
UE MTU	1500 bytes
UE Tx-Power	10 dBm
UE Max-Power	17 dBm
UE Noise Figure	9 dB
LTE Loss Model	HybridBuildingsPropagation

**Table 2:** Wi-Fi Simulation Parameters.

Wi-Fi Parameter	Value
Wi-Fi Standard	802.11a
Wi-Fi Rate Manager	AarfWiFi Rate Manager
Wi-Fi Model	YansWiFi
Channel Width	20 MHz
Wi-Fi TX-Power level	30 dbm
Wi-Fi RX-sensitivity	−101 dBm

**Table 3:** Setup 1 uplink RTT for 20 MB flows.

RTT (ms)	OLIA				LIA				Cubic				wVegas			
	**TB LTE**	**Sim LTE**	**TB Wi-Fi**	**Sim Wi-Fi**	**TB LTE**	**Sim LTE**	**TB Wi-Fi**	**Sim Wi-Fi**	**TB LTE**	**Sim LTE**	**TB Wi-Fi**	**Sim Wi-Fi**	**TB LTE**	**Sim LTE**	**TB Wi-Fi**	**Sim Wi-Fi**
**Average**	2.18	1.14	2.09	0.83	2.47	1.36	1.98	0.73	2.24	1.045	2.049	0.64	2.79	1.57	3.16	3.15
**Std**	0.66	0.058	0.18	0.44	0.25	0.17	0.12	0.21	0.49	0.24	0.43	0.38	0.52	0.44	0.32	0.30

**Table 4:** Setup 1 uplink RTT for 75 MB flows.

RTT (ms)	OLIA				LIA				Cubic				wVegas			
	**TB LTE**	**Sim LTE**	**TB Wi-Fi**	**Sim Wi-Fi**	**TB LTE**	**Sim LTE**	**TB Wi-Fi**	**Sim Wi-Fi**	**TB LTE**	**Sim LTE**	**TB Wi-Fi**	**Sim Wi-Fi**	**TB LTE**	**Sim LTE**	**TB Wi-Fi**	**Sim Wi-Fi**
**Average**	2.94	1.35	2.50	1.87	3.07	1.316	1.741	2.72	2.93	1.240	2.86	1.35	2.86	3.50	4.31	1.96
**Std**	0.36	0.29	0.24	0.15	0.51	0.10	0.29	0.51	0.26	0.04	0.47	0.09	0.49	0.63	0.27	0.32

**Table 5:** Setup 2 uplink RTT for 20 MB flows.

RTT (ms)	OLIA				LIA				Cubic				wVegas			
	**TB LTE**	**Sim LTE**	**TB Wi-Fi**	**Sim Wi-Fi**	**TB LTE**	**Sim LTE**	**TB Wi-Fi**	**Sim Wi-Fi**	**TB LTE**	**Sim LTE**	**TB Wi-Fi**	**Sim Wi-Fi**	**TB LTE**	**Sim LTE**	**TB Wi-Fi**	**Sim Wi-Fi**
**Average**	2.21	1.16	0.23	4.97	2.56	1.16	0.68	3.65	2.04	1.44	0.485	4.62	3.03	4.34	1.09	0.78
**Std**	0.17	0.08	0.10	1.17	0.41	0.11	0.46	1.04	0.48	0.42	0.28	0.99	1.09	0.22	0.35	0.53

**Table 6:** Setup 2 uplink RTT for 75 MB data flows.

RTT (ms)	OLIA				LIA				Cubic				wVegas			
	**TB LTE**	**Sim LTE**	**TB Wi-Fi**	**Sim Wi-Fi**	**TB LTE**	**Sim LTE**	**TB Wi-Fi**	**Sim Wi-Fi**	**TB LTE**	**Sim LTE**	**TB Wi-Fi**	**Sim Wi-Fi**	**TB LTE**	**Sim LTE**	**TB Wi-Fi**	**Sim Wi-Fi**
**Average**	3.13	1.76	18.77	18.88	3.45	1.99	19.35	19.83	3.08	1.21	16.54	18.24	3.94	3.05	20.43	19.98
**Std**	1.94	1.29	2.52	2.54	1.5	1.14	2.12	1.30	1.3	0.11	0.91	2.47	0.97	0.53	0.88	0.75
